# Antiviral Therapy in Chronic Hepatitis C Virus-related Decompensated Liver Cirrhosis – A Tightrope Walk

**DOI:** 10.4103/1319-3767.70606

**Published:** 2010-10

**Authors:** Deepak N. Amarapurkar

**Affiliations:** Department of Gastroenterology, Bombay Hospital and Medical Research Centre, Mumbai, India

Hepatitis C virus (HCV) is a common cause of liver disease leading to liver cirrhosis, liver failure and hepatocellular carcinoma (HCC). Worldwide, HCV-related end-stage liver disease is the most common indication for liver transplantation. It is estimated that 170 million people are infected worldwide. Prevalence of HCV infection is reported to be 12% in some of the centers in the Middle East and 32.3 million HCV infected patients are found in South East Asia and the Middle East.[1] One third of the HCV infected patients are likely to develop liver cirrhosis and complications leading to significant morbidity, mortality and need for liver transplantation. Use of pegylated interferon and ribavirin is the standard care for the treatment of acute and chronic HCV infection without decompensation.[2,3] Decompensated patients with chronic HCV patients need to undergo liver transplantation. Patients who undergo liver transplantation for HCV related decompensated liver disease or HCC are likely to have recurrence of HCV infection universally. Currently published Asian Pacific Association of Study of Liver (APASL) guidelines in 2007[2] recommend that patients with decompensated cirrhosis due to HCV should not be treated with current therapy in general setting and should be referred for liver transplantation. The more recent American Association of Study of Liver (AASLD) guidelines[3] recommend that patients with HCV-related decompensated cirrhosis should be referred for liver transplantation. Interferon-based therapy may be started in low dose and in escalating manner in decompensated cirrhosis, but administering this treatment needs an experienced clinician with vigilant monitoring, for any adverse event, preferably in patients who are already listed for transplantation. Growth factors can be used for treatment associated anemia and leucopenia to improve quality of life and limit the need of antiviral dose reduction.

HCV-related decompensated cirrhosis leads to mortality in more than 50% of cases, by the end of five years without liver transplantation. Primarily, these patients need to be treated for slowing down the disease progression improving synthetic function of liver, reversing complications, decreasing the need for transplantation and preventing relapse of HCV post transplantation. The major concern in interferon-based therapy in these patients is the issue of safety of the treatment. Interferon produces bone marrow suppression that increases the chance of systemic infection worsening anemia and liver function. Some of the complications may be even life threatening. Growth factors have been used to combat cytopenias in these patients. Patients with decompensated cirrhosis in genotype 1 infection have much lower rates of sustained virological response.[4] Till date, 10 trials have been reported for the treatment of decompensated cirrhosis due to HCV infection. Six trials have used interferon and ± ribavirin while four trials have used pegylated interferon plus ribavirin.[5,6] Total number of patients treated were 391. Sustained virological response varied from 0 to 38%, with the highest response rate being reported by us in a genotype 3 predominant population.[7] Treatment discontinuation was reported in 20-50% cases. Fatal complications were also reported in these studies.[5] All these results suggest that sub-set of patients with HCV related decompensated disease may be good candidates for antiviral treatment. The patients who are likely to benefit by antiviral treatment are patients with Model for End-stage Liver Disease (MELD) score ≤ 18, candidates for living donor transplantation, patients who have got a MELD upgrade due to HCC post transplant recurrence can be reduced if pre transplant HCV eradication has been achieved.[5,6] Recently presented data on pegylated interferon and ribavirin therapy prior to living donor transplantation suggested that longer pretransplant therapy was associated with higher post transplant virological response rate, though overall efficacy rate of pegylated interferon and ribavirin were limited and serious adverse events were seen in 54% of the patients.[8] Currently available treatment for decompensated disease with chronic HCV infection has modest efficacy, significant complication rate and needs expert supervision to prevent mortality due to complications. Many novel agents like polymerase and protease inhibitors are in the pipeline for the management of HCV infection. Unfortunately, none of them have been shown to be effective without interferon and ribavirin. In future, combination of these agents may be able to bring down the viremia and help the patients of decompensated liver disease due to HCV infection. In this issue of the Journal, Danish *et al*.[9] review the literature on antiviral therapy in HCV infected decompensated cirrhotics and give some recommendations for managing these patients which will be helpful to the physicians involved in managing such patients.[10]
